# A "late-but-fitter revertant cell" explains the high frequency of revertant mosaicism in epidermolysis bullosa

**DOI:** 10.1371/journal.pone.0192994

**Published:** 2018-02-22

**Authors:** Peter C. van den Akker, Anna M. G. Pasmooij, Hans Joenje, Robert M. W. Hofstra, Gerard J. te Meerman, Marcel F. Jonkman

**Affiliations:** 1 University of Groningen, University Medical Center Groningen, Department of Genetics, Groningen, the Netherlands; 2 University of Groningen, University Medical Center Groningen, Department of Dermatology, Groningen, the Netherlands; 3 Department of Clinical Genetics and the Cancer Center Amsterdam/VUmc Institute for Cancer and Immunology, VU University Medical Center, Amsterdam, the Netherlands; 4 Department of Clinical Genetics, Erasmus Medical Center, Rotterdam, the Netherlands; Centro Nacional de Investigaciones Oncologicas, SPAIN

## Abstract

Revertant mosaicism, or “natural gene therapy”, is the phenomenon in which germline mutations are corrected by somatic events. In recent years, revertant mosaicism has been identified in all major types of epidermolysis bullosa, the group of heritable blistering disorders caused by mutations in the genes encoding epidermal adhesion proteins. Moreover, revertant mosaicism appears to be present in all patients with a specific subtype of recessive epidermolysis bullosa. We therefore hypothesized that revertant mosaicism should be expected at least in all patients with recessive forms of epidermolysis bullosa. Naturally corrected, patient-own cells are of extreme interest for their promising therapeutic potential, and their presence in all patients would open exciting, new treatment perspectives to those patients. To test our hypothesis, we determined the probability that single nucleotide reversions occur in patients’ skin using a mathematical developmental model. According to our model, reverse mutations are expected to occur frequently (estimated 216x) in each patient’s skin. Reverse mutations should, however, occur early in embryogenesis to be able to drive the emergence of recognizable revertant patches, which is expected to occur in only one per ~10,000 patients. This underestimate, compared to our clinical observations, can be explained by the “late-but-fitter revertant cell” hypothesis: reverse mutations arise at later stages of development, but provide revertant cells with a selective growth advantage *in vivo* that drives the development of recognizable healthy skin patches. Our results can be extrapolated to any other organ with stem cell division numbers comparable to skin, which may offer novel future therapeutic options for other genetic conditions if these revertant cells can be identified and isolated.

## Introduction

Revertant mosaicism (RM), or “natural gene therapy”, is the phenomenon in which the effect of germline mutations is corrected by somatic mutational events, and hence constitutes a modifier of disease. RM was first reported in Lesch-Nyhan syndrome in 1988 [1], and subsequently in several other genetic syndromes [2,3]. In 1997, RM was first reported in a genetic skin condition, epidermolysis bullosa (EB) [4], the group of heritable blistering disorders caused by mutations in the genes encoding the components of the epidermal-dermal adhesion complex [5]. While long considered an extraordinary phenomenon, RM has been identified in all major types of EB in recent years (Table 1) [4–19]. Moreover, in a Dutch study RM appeared to be present in all patients with the generalized intermediate subtype of junctional EB (formerly: non-Herlitz junctional EB) on clinical examination, and could be proven at the DNA level in 60% of patients with this EB type [11]. RM has also been shown to be an important disease-modifier in ichthyosis with confetti, another genetic skin disorder, caused by mutations in the *KRT1* or *KRT10* genes [20,21]. These findings have led to the conclusion that, instead of being extraordinary, RM seems to be rather common in EB and led us to hypothesize that RM is present in all patients with EB. In the light of the exciting progress made on revertant cell therapy in recent years [22], boosted especially by the combination with the induced pluripotent stem cell approach [23], as well as the recent successful regeneration of an entire human epidermis from exogenously corrected epidermal stem cells [24], the presence of RM in all EB patients would have important implications for future revertant cell therapy development. In this study, we therefore sought to obtain proof for our hypothesis by employing a mathematical developmental model of the skin. Our results indicate that revertant cells should be present in the skin of all EB patients, but they need a significant selective growth advantage to be able to grow out to clinically recognizable healthy skin patches.

**Table 1:** Revertant mosaicism in epidermolysis bullosa.

Major EB type	EB subtype	OMIM	Corrected gene	OMIM	References
EB simplex	EBS, generalized severe	131760	*KRT14*	148066	[6]
	EBS, autosomal recessive K14	601001	*KRT14*	148066	[7]
Junctional EB	JEB, generalized intermediate (AR)	226650	*COL17A1*	113811	[4,8–12]
			*LAMB3*	150310	[13]
Dystrophic EB	Recessive DEB, generalized severe	226600	*COL7A1*	120120	[14–16]
	Recessive DEB, generalized intermediate	226600	*COL7A1*	120120	[16,17]]
	Dominant DEB	131750	*COL7A1*	120120	[16]
Kindler syndrome	-	173650	*FERMT1*	607900	[18,19]

EB, epidermolysis bullosa; EBS, epidermolysis bullosa simplex; JEB, junctional epidermolysis bullosa; DEB, dystrophic epidermolysis bullosa; AR, autosomal recessive.

## Results and discussion

We used a simplified mathematical developmental model, in which we focused on single nucleotide corrections of nonsense mutations in recessive types of EB (REB), the dominant type of reversion mutations observed in EB [25]. We also focused on the probability that reverse mutations occur until adulthood is reached, not the probability that reverse mutations occur during adult life, because, as discussed later in more detail, the revertant patches that we observe in EB patients develop before adulthood. In this model, the number of basal keratinocytes (BKs), *n*, starting with a single embryonic ectodermal progenitor cell, increases exponentially in the epidermis, *n* = 2^*y*^, with each generation, *y*. The probability that reverse mutations occur until adulthood then depends on four factors: (1) the total number of BKs in an adult human body (estimated to be 36×10^9^, ~2^35^), (2) the number of mitoses required to obtain this number (36×10^9^–1), (3) the probability of a nucleotide alteration per nucleotide per mitosis (~1×10^−9^) [26–28], and (4) the number of target nucleotides that are able to correct the germline mutation when altered (assumed 6 in a recessive model, ignoring the possibility of a nonsense codon being changed into one of the other two nonsense codons). See Table 2 and Methods section at the end of this manuscript for details on the mathematical calculations and quantitative estimates used in this study.[29–35] Our model shows that, during the total number of mitoses, the probability *P* that at least one reverse mutation occurs approaches 1 and reverse mutations are expected to occur 216 times in an average adult human body. This indicates that, indeed, the occurrence of reverse mutations should not be considered extraordinary, but rather an event that can be expected with mathematical certainty in REB patients’ skin carrying mutations that are correctable by single nucleotide mutations. This finding corresponds well to the results of a recent study that demonstrated a strong correlation between the high population incidence of basal cell carcinomas and the high number of basal stem cell mitoses in the skin, which was attributed to stochastic events of numerous randomly occurring somatic mutations [36].

**Table 2:** Quantitative estimates and calculated values in our developmental model of the skin.

Quantitative values		Explanation	References
6 complexes		Average number of rete ridges-dermal papillae complexes per mm skin length	[29]
0.070 mm		Average height of rete ridges-dermal papillae complexes	[30]
1.348 mm		DEJ length corresponding to 1 mm of skin length	Calculated in this study
1.817 mm^2^		DEJ surface corresponding to 1 mm^2^ of skin surface	Calculated in this study
81.7%		Increase in dermo-epidermal contact due to rete ridges-dermal papillae complexes	Calculated in this study
10 μm		Diameter of circular base of basal keratinocytes	[31]
20,000 cells		Number of basal keratinocytes per mm^2^ skin	Used in this study
	15,000 cells	Number of basal keratinocytes per mm^2^ skin	[32]
	20,000–30,000 cells	Number of basal keratinocytes per mm^2^ skin	[33]
	23,146 cells	Number of basal keratinocytes per mm^2^ skin	Calculated in this study
1.8 m^2^		Average skin surface in adult human body	Used in this study
3.3x10^6^ mm^2^		Average total DEJ surface in adult human body	Calculated in this study
36x10^9^ cells		Average total number of basal keratinocytes in adult human body	Calculated in this study
(36x10^9^–1) mitoses		Number of mitoses needed to obtain total number of basal keratinocytes	Calculated in this study
35.1 generations		Number of generations of cells needed to obtain total number of basal keratinocytes	Calculated in this study
1x10^-9^		Approximate per nucleotide point mutation rate per mitosis	[26–28]
6x10^9^ nucleotides		Approximate number of nucleotides per genome	[34]
6 mutations		Expected number of novel point mutations per mitosis	Calculated in this study
216x10^9^ mutations		Expected number of point mutations in basal keratinocytes of human body at adulthood	Calculated in this study
36x		Average point mutation frequency of each nucleotide in basal keratinocytes of human body at adulthood	Calculated in this study
105		Total number of somatic mutations expected to have accumulated per BK at adulthood	Calculated in this study
6 nucleotides		Number of target nucleotides for a reverse mutation in REB patients with 2 nonsense mutations	Calculated in this study
6x10^-9^		Probability of a reverse point mutation per mitosis	Calculated in this study
1.6x10^-94^		Probability that no reverse mutation occurs during total (36x10^9^–1) mitoses (*P*_*not*_)	Calculated in this study
~1		Probability that at least one reverse mutation occurs during total number of mitoses (*P* = 1 –*P*_*not*_)	Calculated in this study
216 mutations		Expected number of reverse point mutations during (36x10^9^–1) mitoses	Calculated in this study
100 mm^2^		Minimal size of revertant patch to be clinically recognizable	[9], Fig 1
2x10^6^ cells		Number of basal keratinocytes required for a revertant patch	Calculated in this study
21 generations		Number of generations needed to obtain 2x10^6^ basal keratinocytes	Calculated in this study
14^th^ generation		Generation in which revertant cell should occur to obtain revertant patch of 100 mm^2^ in generation 35	Calculated in this study
0.0001		Probability *P* that at least one reverse mutation occurs in the first 14 generations	Calculated in this study
1/10,000 patients		Number of patients predicted to carry clinically recognizable revertant skin patch	Calculated in this study
1:1,000		Long term proliferating epidermal stem cells:other basal keratinocytes ratio	[24,35]
156		Expected number of reverse mutations in epidermal stem cells during adult life	Calculated in this study

DEJ, dermo-epidermal junction; REB, recessive epidermolysis bullosa

**Fig 1:**
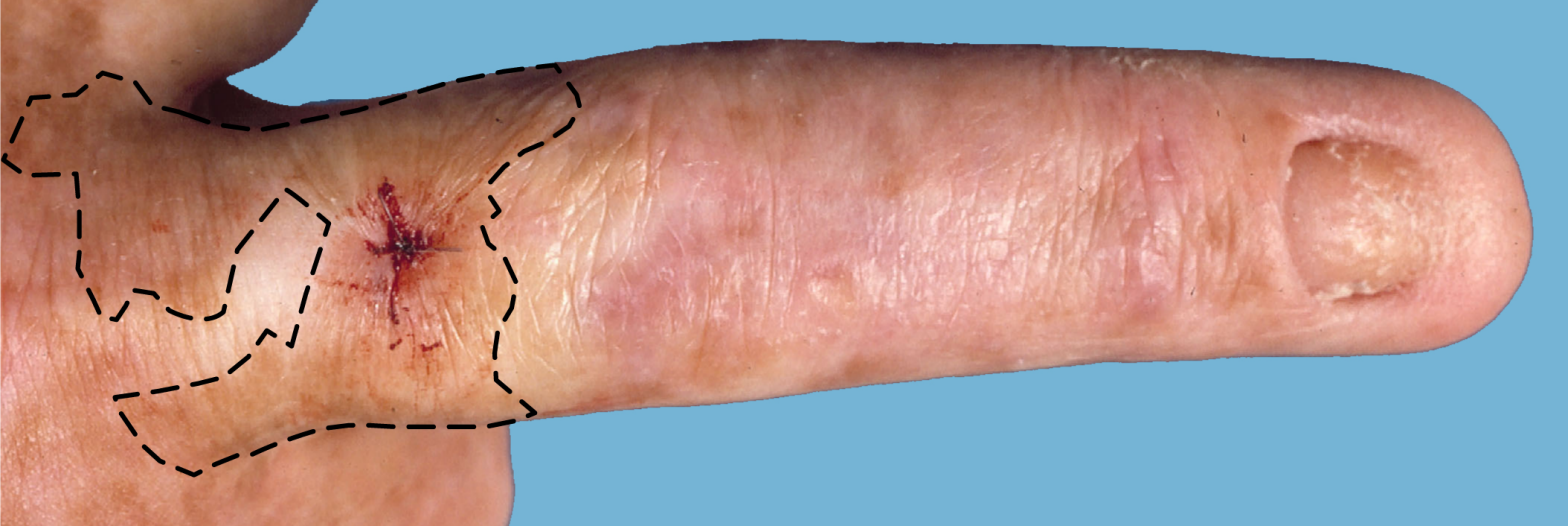
The smallest revertant patch observed in any of our patients. Photograph of the revertant patch on the dorsal middle finger of patient EB093-01 (deceased) who had generalized intermediate junctional EB due to compound heterozygous *COL17A1* mutations c.[3676C>T];[4319dup], p.[Arg1226*];[Gly1441Trpfs*14] [9]. Note stitched biopsy site for confirmation of type XVII collagen re-expression. The size of the patch was approximately 2 cm^2^ and it allowed him to wear a wedding ring. Based on this, we concluded revertant patches should be ~1 cm^2^ minimum in order to be clinically recognizable.

Knowing that revertant mutations should occur frequently in the skin of patients with REB, the next question is whether these revertant cells will be able to grow out to healthy, revertant skin patches that are clinically recognizable. To induce recognizable patches, i.e. patches covering at least 1 cm^2^ (corresponding to 2×10^6^ revertant BKs) (Fig 1) [9], reverse mutations in our model should arise in the 14^th^ cell generation the latest (2^14^ = 16,384 BKs) (Methods section, Table 2). This number of cells is supposed to be reached before the 4^th^ week of embryogenesis [37]. The probability *P* that at least one reverse mutation will have occurred in the first 14 generations is only ~0.0001. A clinically recognizable, healthy revertant skin patch should therefore be expected in only one per ~10,000 patients (~1/0.0001).

Evidently, this conclusion strongly contradicts our clinical observations that revertant patches are present in all Dutch patients with generalized intermediate junctional EB [11]. This underestimate can only be partly explained by the developmental model itself. First, the model ignores cell loss due to normal biologic processes, like apoptosis and differentiation. As the probability that reverse mutations occur depends on the number of mitoses needed to obtain final cell numbers, integrating cell loss will increase this probability by means of increasing the total number of mitoses. To get an impression of the effect of cell loss, we next used a conservative model in which we assumed that 90% of new cells in each generation would be lost, *n*_*y*_ = 1.1^*y*^, as the exact cell loss rate is unknown. In this model 359×10^9^ mitoses (255 generations) are required to obtain the 36×10^9^ BKs of an adult human body; and 19.6×10^6^ mitoses (152 generations) are required for the 2×10^6^ cells of a 1 cm^2^ recognizable revertant patch, assuming identical growth and loss rates for mutant and revertant cells. The reverse mutation should then occur no later than in the 103^rd^ generation, when the absolute minimal number of cells needed to obtain a revertant skin patch at adulthood is reached (18×10^3^ BKs, 180×10^3^ mitoses), which again is before the 4^th^ week of embryonic development [37]. Loss of 90% of cells in each generation would thus increase the required number of mitoses ~10x compared to the initial model. The probability of recognizable patches (*P* = 0.0011) consequently also increases a factor 10. Hence, even with 90% cell loss in each generation, recognizable revertant patches could be expected in only one per ~909 patients (~1/0.0011) and cannot explain their much more frequent clinical appearance.

Second, we assumed that all BKs are equal and need to be revertant. In reality, a hierarchy among BKs is recognized: long-term proliferating epidermal stem cells produce clonogenic transient amplifying cells that provide the other (transient amplifying) BKs to the epidermal proliferating units, which collectively form skin patches [35,38]. Only a limited number of epidermal stem cells thus need to be revertant in order to acquire recognizable revertant patches. This critical number of revertant stem cells per mm^2^ needed for recognizable patches is unknown, but it is undoubtedly smaller than the number used before. To estimate the possible effect of BK hierarchy, we assumed an epidermal stem cell:non-stem cell BK ratio of 1:1,000, based on the review by Strachan and Ghadially [35] and the recent experimental study by Hirsch et al. [24], who showed that the number of stem cells in their skin transplants was approximately 1.8×10^3^ per cm^2^, i.e. approximately 1:1,000 to the number of 2×10^6^ BKs that we calculated to be present per cm^2^ skin. This reduces the number of mitoses in which reverse mutations can occur 1,000-fold. Consequently, the probability *P* that at least one reverse mutation occurs decreases substantially to 0.19 (as compared to ~1 in the initial model). As the ratio of cells in the entire body skin vs. a minimal recognizable patch will be left unchanged—both are reduced 1,000-fold by considering epidermal stem cells—the probability of a recognizable patch will also be left unchanged, i.e. one per ~10,000 patients. In other studies, the stem cell:non-stem cell BKs ratio has been estimated to be up to 1:10 [36,39], but even if stem cell numbers in the skin are this high, the probability of the occurrence of recognizable revertant skin patches would not the altered. Hence, integrating stem cells in the model cannot explain the discrepancy between our clinical observations and the predictions following from the model.

Third, we used the average human somatic cell mutation rate (1×10^−9^/nt/mitosis) [27], as data on the actual mutation rate for BKs are not available, which could be substantially higher. However, only a ~100,000-fold increase in mutation rate to at least 1×10^−4^ per nucleotide per mitosis could explain the occurrence of reverse mutations in the 14^th^ cell generation in every patient. A recent study performing ultra-deep sequencing of 74 cancer genes in 234 normal sun-exposed skin biopsies from human eyelids identified 2–6 somatic mutation per megabase per cell (12,000–36,000 mutations per diploid genome) [40]. We calculated the average number of somatic mutations to be approximately 105 per BK at adulthood (see Methods section). Assuming these BKs self-renew monthly to maintain homeostasis for 60 years (720 mitoses), this would accumulate to approximately 4,425 somatic mutations in BKs in aged skin. Although the data by Martincorena et al. might thus suggest a higher mutation rate in human skin than the average human mutation rate, most mutations in their study had a UV-signature, indicating that such a higher mutation rate is likely attributable to environmental factors rather than to an intrinsically higher mutation rate. In addition, their results were obtained on entire epidermis, making it hard to compare to our calculations for BKs only. One could obviously argue that environmental factors play a major role in the occurrence of reverse mutations in human skin and indeed UV-exposure might induce reverse mutations during life. Interestingly though, many reverse mutations do not have a UV-signature [11], reducing the probability that they occur due to environmental factors and supporting our hypothesis that they should arise early during embryonic development.

**Fig 2:**
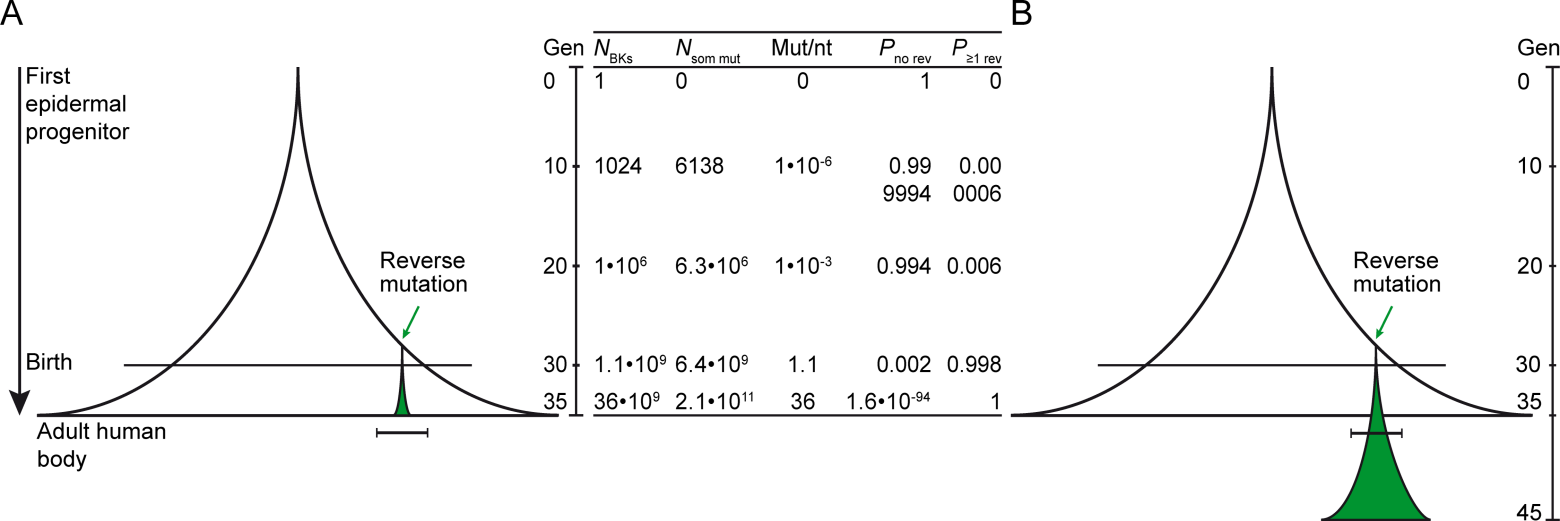
Selective growth advantage may explain the occurrence of clinically recognizable revertant patches. (A) Schematic representation of the “bell-shaped” increase in cell number according to the *n = 2*^*y*^ model starting with a single progenitor. In the later generations, the probability of a reverse mutation approaches 1 asymptotically. However, a reverse mutation that occurs in later generations cannot result in a clinically recognizable patch since the revertant cell cannot go through the required number of mitoses. (B) We therefore propose that revertant cells have a selective growth advantage, e.g. they possess the ability to go through more generations than their mutant neighbors. This would allow reverse mutations to occur at later stages and still result in visible patches. Green area: revertant area. Horizontal bar: size of minimal clinically recognizable patch (≥ 1 cm^2^). Gen, generation of cells. *N*_BKs_, number of basal keratinocytes. *N*_som mut_, expected total number of new somatic mutations. Mut/Nt, average number of mutations per nucleotide. *P*_no rev_, probability of no reverse mutation. *P*_≥1 rev_, probability of at least one reverse mutation.

The three points mentioned above may explain a small portion of the discrepancy between our clinical observations and the predictions from the model. However, another, more likely explanation why revertant cells grow out to recognizable patches in more-than-expected patient numbers is that revertant cells possess a strong selective growth advantage over their non-revertant neighbors, equivalent to what is seen for tumor cells carrying driver mutations in certain cancer-related genes [40]. If revertant cells are able to survive longer or to go through more cycles of cell divisions, reverse mutations would be allowed to occur at later stages of development, when their occurrence is more likely (Fig 2). In hematologic diseases involving hematopoietic bone marrow stem cells, such a growth advantage is believed to explain the ability to detect reverse mutations and clinical improvement in the first place [41–46]. As skin is also a continuously self-renewing organ, similar mechanisms may be active in skin as in the bone marrow. Additionally, growth potential differences between wild-type and mutant cell lineages have been shown for other diseases like Wiskott-Aldrich syndrome and neurofibromatosis type I [47,48].

**Fig 3:**
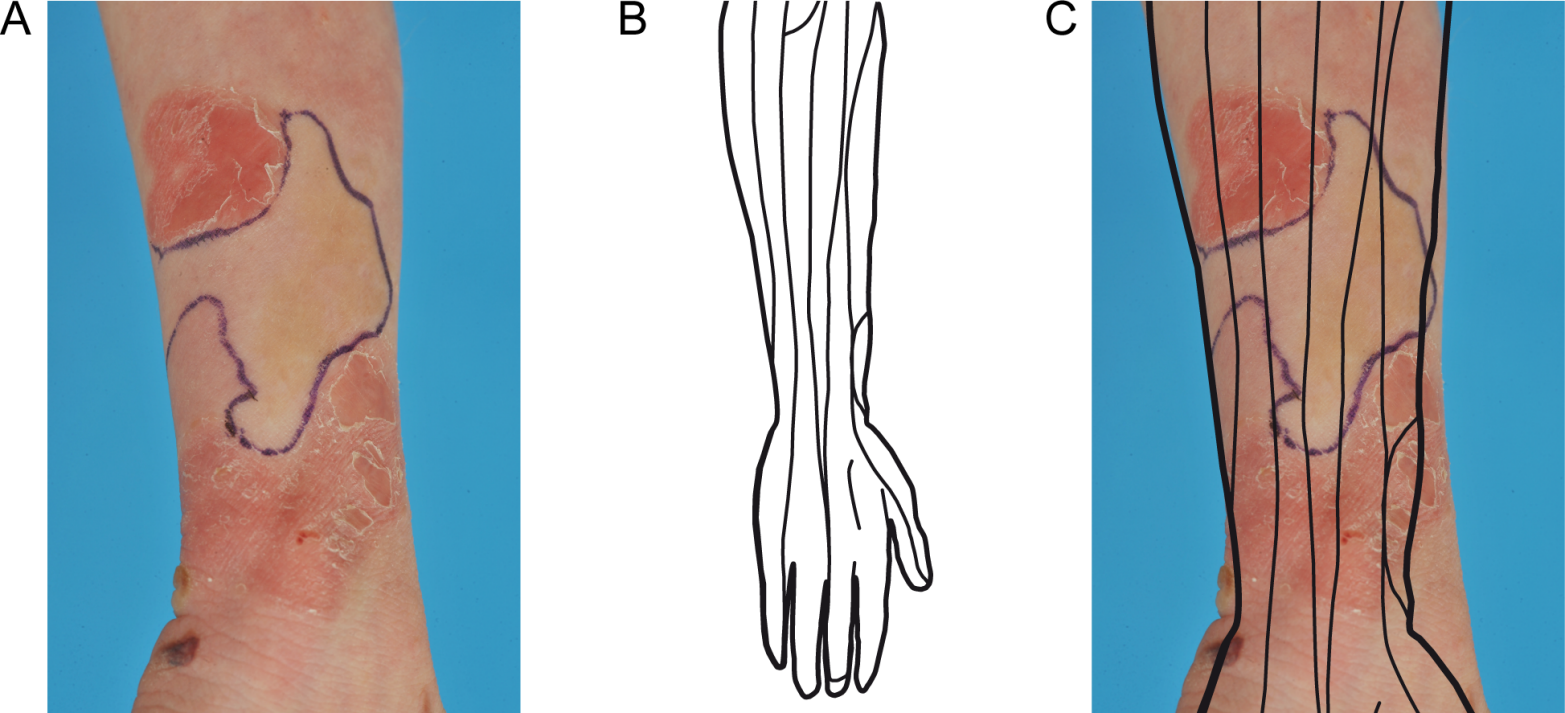
A selective growth advantage of revertant cells is supported by the patterns of the revertant skin patches. (A) Dorsal forearm of an 8-year old boy (EB134-01) with generalized intermediate junctional EB due to the compound heterozygous *COL17A1* mutations c.[1260del];[3496_3497del], p.[Thr421Leufs*72];[Ser1166Leufs*6] showing a revertant patch (indicated by blue line). (B) The lines of Blaschko on the dorsal forearm as deduced by Blaschko and Happle [49,50]. (C) The lines of Blaschko projected on the revertant patch on the dorsal forearm of patient EB134-01. The revertant patch has clearly exceeded the boundaries of the Blaschko lines and has grown into adjacent Blaschko line segments. The parents of patient EB134-01 have given written informed consent (as outlined in PLOS consent form) to publish this photograph.

Several arguments support a selective growth advantage of revertant cells in REB skin. First, revertant patches are not confined to the lines of epidermal development, the lines of Blaschko [49,50]. Rather, they appear to develop in one Blaschko-line segment and then expand centrifugally into adjacent segments (Fig 3). This observation indicates that reverse mutations occur when epidermal formation from the Blaschko-lines is completed, i.e. after the end of the 4^th^ week [37]. This is in line with our conclusion that reverse mutations likely occur in later stages when the number of epidermal cells has reached the critical amount to allow their occurrence with a higher probability.

Second, a segmental phenotype due to somatic second-hit mutations has not been reported in carriers of REB mutations. As there are many more target nucleotides for gene silencing than for mutation correction, the probability that the wild-type allele is silenced in carriers far exceeds the probability of reverse mutations in REB patients’ skin [27]. Although carriers may not all have been meticulously evaluated for small areas of increased skin blistering propensity, the lack of reports on a segmental phenotype in carriers suggests a significant growth disadvantage of cells harboring second-hit mutations.

Third, the expanding revertant patches seen in ichthyosis with confetti due to multiple correction events in either the *KRT1* or *KRT10* genes seem to support this hypothesis of a growth advantage or greater fitness of corrected skin cells over their mutant neighbors [20,21,51,52]. While normal epidermal stem cell units are considered to colonize up to 3 mm in diameter [35], patches in ichthyosis with confetti due to *KRT10* mutations, which first appear around the age of 6 years, are reported to grow up to 4 cm in diameter [20].

In our model, the 29^th^ cell generation is the first where the number of mitoses is so high that more than one reverse mutation is expected to occur. Revertant cells need another 21 generations to acquire the 2×10^6^ cells of a minimal recognizable patch. They would thus need to go through at least 15 (21 vs. 6) more generations than their mutant neighbors. Future studies, perhaps using *in vivo* imaging techniques in new luminescent and fluorescent genetic animal models [53], need to clarify whether the revertant growth advantage is indeed of this order of magnitude. Two different mechanisms may act synergistically in providing the enormous revertant growth advantage: first, revertant cells may survive longer and, second, may possess the ability to go through more cell divisions.

Despite their likely growth advantage, the majority of patients do not report expansion of revertant patches in adulthood, and existing revertant patches do not expand into blistered areas at their borders. This suggests a limited time frame where the growth advantage can be exerted, with an end point before adulthood. In a child with Kindler syndrome the revertant patch expanded relatively to body size until the age of 8 years [18]. This indicates that revertant patches may have the ability to expand at least until this age. Similar observations have been made in ichthyosis with confetti (IWC type I) due to *KRT10* frameshift mutations, in which multiple revertant patches arise during childhood and stop expanding before adulthood [20]. In IWC type II caused by *KRT1* frameshift mutations, on the other hand, patches usually arise at a later age [21], indicating that the time window of revertant growth advantage is limited, but likely varies per gene or even mutation.

Why revertant patches lose their ability to expand further after a certain time point and are not able to cover the entire body with new, healthy skin and thus cure the disease, is unknown. In the study by Hirsch et al., exogenously corrected epidermal stem cells were cultured to produce 0.85 m^2^ of new epidermis to treat a child with junctional EB due to a homozygous *LAMB3* mutation [24]. From this study, it would be expected that corrected stem cells would have the ability to expand *in vivo* as well, but this is not what is seen in adult EB patients. In their review, Colom and Jones state that cells with carcinogenic driver mutations likely have a short-term advantage over their neighbors, after which their growth is constrained and they revert to homeostatic behavior. If this is true, a similar mechanism could be active in revertant cells [54]. Also, age-related changes in the balance between proliferation and differentiation gene expression programs, for instance due to epigenetic changes at the epidermal differentiation complex locus (1q21) or of proliferation and differentiation gene networks [55–58], could play a role. Further studies comparing the genomic and epigenomic constitution of revertant and non-revertant cell lineages could help solve this issue.

In addition to existing patches not expanding further after a certain age, we have neither witnessed the occurrence of new revertant patches in adult EB patients nor has this been reported in the literature. Of note, one patient claimed that his revertant patch had occurred “during life”, but long-term photographic could only confirm the presence of the same patch throughout follow-up, leaving the question unanswered whether this is a true example of a new revertant patch occurring later in life [13]. This is particularly remarkable when the occurrence of new mutations during adulthood is considered (see Methods section). Analogous to the calculations used to obtain the frequency of reverse mutations during embryonic development until adulthood, it can be estimated that at least 156 new reverse mutations will occur in epidermal stem cells alone during adult life (Table 2). The lack of new revertant patches arising during adulthood thus supports the hypothesis that the growth advantage of revertant cells decreases during life and has a limited time window.

Altogether, our developmental model predicts that REB patients with mutations that are correctable by single point reversions carry numerous reverse mutations in their skin. Our observation of revertant patches in at least all the patients with one junctional REB subtype seems to form the clinical proof for this prediction. The question remains as to whether it is also true in other REB subtypes, but this prediction should encourage clinicians to scrutinize every EB patient for revertant patches (i.e. by asking the patients about the presence of skin areas that never blister, clinical examination and a ballpoint rub test on suspected patches [16]), as their presence offers exciting future therapeutic prospects [22,23]. To produce clinically recognizable revertant patches, reverse mutations should, however, arise so early during embryogenesis that that is expected to occur in only one per ~10,000 patients. We therefore postulate the “late-but-fitter revertant cell” hypothesis: reverse mutations arise at later stages of development, but confer a strong selective growth advantage to the revertant cells that drives their development into revertant patches. Our results indicate that revertant cells should also be present in the affected tissues in other genetic diseases, provided their stem cell division numbers are comparable to skin (and bone marrow), like the colorectal tissues [36]. Table 3 shows all genetic conditions in which RM has been identified to date [1,20,21,41,46,59–75]. Next generation sequencing techniques have opened unprecedented opportunities for the detection of low-grade somatic mosaicism and are perfectly suited to prove whether this hypothesis is indeed true [76]. If ways can be found to subsequently identify and isolate these cells, this could offer novel opportunities for revertant cell therapy in other genetic diseases too.

**Table 3:** Revertant mosaicism in genetic diseases other than epidermolysis bullosa.

Disease	OMIM	Corrected gene	OMIM	References
Lesch-Nyhan syndrome	300322	*HPRT*	308000	[1]
Ichthyosis with confetti, KRT10	609165	*KRT10*	148080	[20]
Ichthyosis with confetti, KRT1	609165	*KRT1*	139350	[21]
Fanconi anemia, complementation group A	227650	*FANCA*	607139	[41]
Dyskeratosis congenita type 1	127550	*TERC*	602322	[46]
Duchenne muscular dystrophy	310200	*DMD*	300377	[59]
Myotonic dystrophy	160900	*DMPK*	605377	[60]
Tyrosinemia type I	276700	*FAH*	613871	[61]
Bloom syndrome	210900	*RECQL3*	604610	[62]
Adenosine deaminase deficiency	102700	*ADA*	608958	[63]
Hereditary motor and sensory neuropathy type 1A	118220	*PMP22 duplication*	601097	[64]
X-linked severe combined immunodeficiency	300400	*IL2RG*	308380	[65]
Fanconi anemia, complementation group C	227645	*FANCC*	613899	[66]
Wiskott-Aldrich syndrome	301000	*WAS*	300392	[67]
X-linked hypohidrotic ectodermal dysplasia with immunodeficiency	300291	*NEMO*	300248	[68]
Omenn syndrome	603554	*RAG1*	179615	[69]
T-cell immunodeficiency	610163	*CD3-zeta (CD247)*	186780	[70]
Fanconi anemia, complementation group N	610832	*PALB2*	610355	[71]
Fanconi anemia, complementation group I	609053	*FANCI*	611360	[72]
Leukocyte adhesion deficiency type 1	116920	*ITGB2*	600065	[73]
Autosomal recessive severe combined immunodeficiency	600802	*JAK3*	600173	[74]
Autosomal recessive severe combined immunodeficiency	608971	*IL7R*	146661	[75]

## Methods

### Determining the occurrence of reverse mutations in basal keratinocytes

We have used an exponential model in which the total number of basal keratinocytes (BKs), *n*, starting with a single embryonic ectodermal progenitor cell, doubles with every generation of cells, *y*, according to *n = 2*^*y*^. The total number of BKs, *n*_*total*_, in an average adult human body is a function of the total body dermo-epidermal junction (DEJ) surface *S*_*DEJ*_ and the number of BKs per unit DEJ. The *S*_*DEJ*_ is not identical to the human skin surface *S*_*skin*_, as it is greatly increased by the presence of rete ridges and dermal papillae. Assuming an average of 6 rete ridges-dermal papillae complexes per mm skin length (*L*_*skin*_) with an average height of 0.070 mm [28,29], the DEJ can be described as a sinus function with amplitude 0.035 mm and period 12*π* (Table 2):

f(x)=0.035∙sin⁡(12∙π∙x)

The exact length of the line described by this sinus, i.e. the actual DEJ length *L*_*DEJ*_, is calculated by solving the integral of the function (Mathematica, Wolfram Research Inc., Champaign, IL)

$$L_{DEJ}=F(x)=\int_{a}^{b}\sqrt{1+(0.42∙\pi ∙\cos(12∙\pi ∙x){)}^{2}}dx$$

Using *a* = 0 and *b* = 1 mm *L*_*skin*_ reveals that 1 mm of *L*_*skin*_ corresponds to 1.348 mm *L*_*DEJ*_. Consequently, since the “finger like appearance” of dermal papillae resembles two interfering sinus waves [77], 1 mm^2^ *S*_*skin*_ corresponds to (1.348)^2^ ≈ 1.817 mm^2^ *S*_*DEJ*_. Rete ridges thus theoretically expand the dermal-epidermal surface contact by 81.7%.

The number of BKs was measured to be between 15,000 and 20,000 to 30,000 per mm^2^ skin [33,33]. Assuming that BKs have a circular base of diameter ~10 μm [30] and surface ~78.5 μm^2^, approximately 12,739 BKs are expected per mm^2^ *S*_*DEJ*_ (1 mm^2^ *S*_*DEJ*_ / 78.5×10^−6^ mm^2^ per BK) and 23,146 BKs per mm^2^ *S*_*skin*_. Taken together, we decided to use 20,000 BKs per mm *S*_*skin*_ in further calculations.

Assuming a total human adult body surface of 1.8 m^2^ (corresponding to 3.3×10^6^ mm^2^ *S*_*DEJ*_) the total number of BKs in an adult human body *n*_*total*_ *=* 1.8×10^6^ mm^2^ × 20,000 BKs/mm^2^ = 36×10^9^. This number is reached in the 35^th^ generation, after a total of 36×10^9^–1 mitoses. Given a per nucleotide point mutation rate of approximately 1×10^−9^ per mitosis [26–28] and 6×10^9^ nucleotides per diploid genome [34], ~6 novel point mutations may be expected in the daughter cell’s genome in each cell division. In the 35^th^ generation of cells, the total expected number of new point mutations that have occurred in the body’s BKs is approximately 6 × total number of mitoses = 216×10^9^, indicating that each single nucleotide is expected to be mutated on average 36 times in the human basal skin layer at adulthood. In total, 3.6×10^12^ somatic mutations are expected to have accumulated in the body’s BKs at adulthood, which equates to an average of 105 somatic mutations per BK.

Alteration of one of the nucleotides of a nonsense codon will usually change the nonsense into a sense codon. If nonsense mutations are present on both alleles, there are therefore 6 target nucleotides for a reversion, thereby ignoring the small probability that the nonsense codon will be changed into one of the other two possible nonsense codons. For frame-shift and splice-site mutations, the number of potential target nucleotides for reversion events are usually unknown but theoretically numerous. For missense mutations, this number is probably less than 6. We therefore decided to use the number of 6 target nucleotides of nonsense codons in our calculations. A larger or smaller number of target nucleotides would not affect the order of magnitude of the probability of the occurrence of reverse mutations. The probability of a reverse point mutation per mitosis in case of 6 target nucleotides is thus *p*_*rev*_ = 6 × 1×10^−9^ = 6×10^−9^, The probability that no reverse mutation will occur during the total number of mitoses until the 35^th^ generation (*P*_*not*_) is

$$P_{not}=(1-p_{rev}{)}^{total\;number\;of\;mitoses}={\left(1-6∙10^{-9}\right)}^{\left(36∙10^{-9}-1\right)}=1.6∙10^{-94}$$

Therefore, the probability *P* that at least one reverse mutation occurs during these mitoses is

$$P=1-P_{not}≅1$$

The expected number of reverse mutations in 36×10^9^–1 mitoses is (36×10^9^–1) × 6×10^−9^ = 216.

### Determining the occurrence of clinically recognizable revertant skin patches

One cm^2^ of revertant *S*_*skin*_ corresponds to 100 mm^2^ × 20,000 BKs/mm^2^ = 2×10^6^ revertant BKs. According to the 2^*y*^ model, it takes 21 generations to obtain this number from one single revertant ancestor. This implies that a reverse mutation should have arisen at the latest in the *y* = 35–21 = 14^th^ generation, corresponding to a stage of 2^14^ = 16,384 BKs. Considering that in early embryonic stages rete ridges and dermal papillae are not yet developed [37], the reverse mutation should have arisen at the embryonic stage where the embryo’s body surface was less than 2 mm^2^. For comparison, a 4-week embryo measures approximately 5 mm in length and, if compared to a cylinder with height = 5 mm and radius = 0.5 mm, its body surface is in the order of 17 mm^2^, indicating that a reverse mutation should arise before the 4^th^ week of embryonic development in order to give rise to a clinically recognizable revertant skin patch. The probability *P*_*not*_ that a reverse mutation does not arise in the first 14 generations is

$$P_{not}={\left(1-6∙10^{-9}\right)}^{\left(16,384-1\right)}=0.9999$$

and the probability *P* that at least one reverse mutation arises in the first 14 generations is

P=1−0.9999=0.0001

Therefore, a clinically recognizable revertant skin patch is expected once in only ~1/0.0001 = 10,000 patients.

### Determining the occurrence of reverse mutations in epidermal stem cells during adult life

The number of long-term proliferating epidermal stem cells (ESCs) in an adult human body was estimated earlier to be approximately 36×10^6^ (total number of BKs / 1,000). Assuming every ESC divides asymmetrically every month to maintain homeostasis, analogous to the self-renewal time of the entire epidermis, every ESC would go through 12 × 60 = 720 mitoses until the age of 78 years, totaling to 720 mitoses × 36×10^6^ ESCs = 26×10^9^ mitoses in the entire human body. Assuming the daughter cells would remain as ESCs in the basal layer, 26×10^9^ mitoses × 6 mutations/mitosis = 156×10^9^ new somatic mutations are expected to accumulate, equivalent to 26 mutations per nucleotide (156×10 ^9^ mutations / 6×10^9^ nucleotides per diploid genome). As there are 6 candidate nucleotides for reverse mutations, 6 × 26 = 156 reverse mutations are expected in ESCs during adult life.
